# Kinematic features in patients with lateral discoid meniscus injury during walking

**DOI:** 10.1038/s41598-018-22935-0

**Published:** 2018-03-22

**Authors:** Zefeng Lin, Wenhan Huang, Limin Ma, Lingling Chen, Zhiqiang Huang, Xiaolong Zeng, Hong Xia, Yu Zhang

**Affiliations:** 1 Department of Orthopedics, Guangzhou General Hospital of Guangzhou Military Command, Guangzhou, 510010 China; 2 Guangdong Key Laboratory of Orthopedic Technology and Implant Materials, Guangzhou, 510010 China; 3 Department of Orthopaedics and Traumatology, Prince of Wales Hospital, The Chinese University of Hong Kong, Hong Kong, China; 4 Southern Medical University, Guangzhou, 510515 China; 5 Guangzhou Medical University, Guangzhou, 511436 China

## Abstract

At present, there few studies on the kinematic features of lateral discoid meniscus injury. In this study, a motion capture system was used to investigate the motion characteristics of knees with lateral discoid meniscus after injury, and the differences between the knees with lateral meniscus and intact knees were compared. Fourteen patients diagnosed with unilateral lateral discoid meniscus injury, fourteen patients diagnosed with unilateral lateral meniscus injury, and fourteen normal subjects with healthy knees were recruited and grouped. Through kinematic gait analysis, it was found that the subjects in the two groups with meniscus injuries exhibited significantly smaller ranges of rotation and translation than those with healthy knees on the sagittal, coronal, and horizontal planes, but not in proximal-distal translation. Maximum lateral tibial translation and maximum internal tibial rotation in the knees with lateral discoid meniscus injury were significantly decreased compared to those with lateral meniscus injury. The results show that the kinematic features of knees with lateral discoid meniscus injury are statistically different than those of healthy knees and knees with lateral meniscus injury. This study provides an important reference for the dynamic function of knees with lateral discoid meniscus injury.

## Introduction

The term “discoid meniscus” is considered to be synonymous with “snapping knee syndrome”. Some researchers estimate the incidence of discoid lateral meniscus to be 0.4%–17%, whereas discoid medial meniscus is extremely rare (0.1%–0.3%)^1–3^. However, the true incidence may be much higher because discoid meniscus is usually found after an injury. It is well known that the meniscus plays an important role in knee joint stability because it can distribute loads and, therefore, reduce stresses on the tibia^4^. The anatomical structure of a discoid meniscus is abnormal compared to a normal meniscus. Patients with discoid meniscus have increased risk of sustaining meniscal injuries, which may induce some clinical problems, such as pain, swelling, joint twisting, and motion constraint^5^.

The diagnosis of discoid meniscus is mainly done through magnetic resonance imaging (MRI). Generally, MRI has the advantage of detecting meniscus tears and abnormal shifts^6,7^, but it has been reported that MRI has low sensitivity in diagnosing lateral meniscus in children^8^. The deficiency is due to the difficulty involved in checking the stability of a discoid meniscus by using MRI and the fact that some incomplete discoid menisci can appear normal in MRI^9,10^. Radiographs are commonly used in the diagnosis of patients with discoid meniscus. Although discoid meniscus cannot directly be identified through radiographs, some of its features can be revealed by observing lateral joint space and the appearance of the lateral femoral condyle and lateral tibial plateau^9,10^.

Kinematic analysis plays an important role in the evaluation of knee injuries, and it can provide more dynamic and immediate observational information than static medical imaging^11,12^. The dynamic functions of knees can be objectively and quantitatively evaluated using motion capture systems (optical motion capture or monoplane/biplane fluoroscopy). Traditional motion capture systems have already been used to evaluate the functional motion of the knees on patients with anterior cruciate ligament (ACL) deficiency in clinical studies^13–16^. These studies provided some evidence about how to evaluate clinical outcomes. However, motion capture systems are usually complicated and require many supplementary and auxiliary devices, which greatly limits their clinical application. Thus, it is not practical to introduce these devices to hospitals or clinics. Fluoroscopic motion capturing techniques enable researchers to accurately quantify three-dimensional bone motion during dynamic activities without soft tissue artifact (STA). However, this technology only enables constrained motion patterns to be investigated due to its limited field-of-view. It is not suitable for use in long-term clinical supervision because of X-ray radiation. In recent years, with the algorithm improvements of optical capturing systems and the simplification of their construction, portable motion capture systems have already been used to investigate knee joint functions and evaluate the gait of patients with knee injuries. Portable motion capture systems are easy to operate, and only two operators are required. Therefore, they are likely to be promoted for uses from laboratory investigation to clinical application^13,14,17–19^.

In the current study, the kinematic characteristics of patients with injured lateral discoid meniscus were investigated using a portable infrared motion capture system. The detection of the injured discoid meniscus and the clinical feasibility of the motion capture system are discussed. Based on infrared navigation technology, the infrared induction system was fixed to the body surface, and an induction system was used to reflect the mobile data from the infrared signal gauge points as body motion. Then the motion capture system analyzed the three-dimensional activities of knees and evaluated the kinematics characteristics of knees. This study focused on detecting the gait characteristics of patients with injured lateral discoid meniscus based on infrared navigation technology and comparing the differences among knees with injured lateral discoid meniscus and injured lateral meniscus and healthy ones.

## Results

In the lateral discoid meniscus injury group (Group A) and the normal meniscus injury group (Group B), range of motion (ROM) and six degrees of freedom (DOF) in the knee (exclusive of proximal/distal translation) were considerably decreased compared to those of healthy knee joints (p < 0.05). Among patients with injured meniscus in the lateral compartment (normal morphology and discoid meniscus), there were no significant differences in ROM (Table 1).

**Table 1:** ROM in 6DOF of each group (mean ± SD).

	Group A	Group B	Group C
adduction/abduction (deg.)	5.58 ± 1.17^*^	6.60 ± 1.19^#^	9.89 ± 2.01
internal/external (deg.)	8.35 ± 2.38^*^	8.64 ± 1.51^#^	15.39 ± 2.37
flexion/extension (deg.)	43.05 ± 8.63^*^	41.94 ± 6.40^#^	57.43 ± 6.25
anterior/posterior (mm)	8.28 ± 2.62^*^	9.50 ± 2.02^#^	15.54 ± 3.87
proximal/distal (mm)	11.29 ± 3.78	11.23 ± 3.43	12.42 ± 4.97
medial/lateral (mm)	6.13 ± 1.54^*^	7.12 ± 1.63^#^	10.30 ± 2.93

*P < 0.05 between Group A and Group C.

^#^P < 0.05 between Group B and Group C.

No statistical significance is found between Group A and Group B.

**Figure 1:**
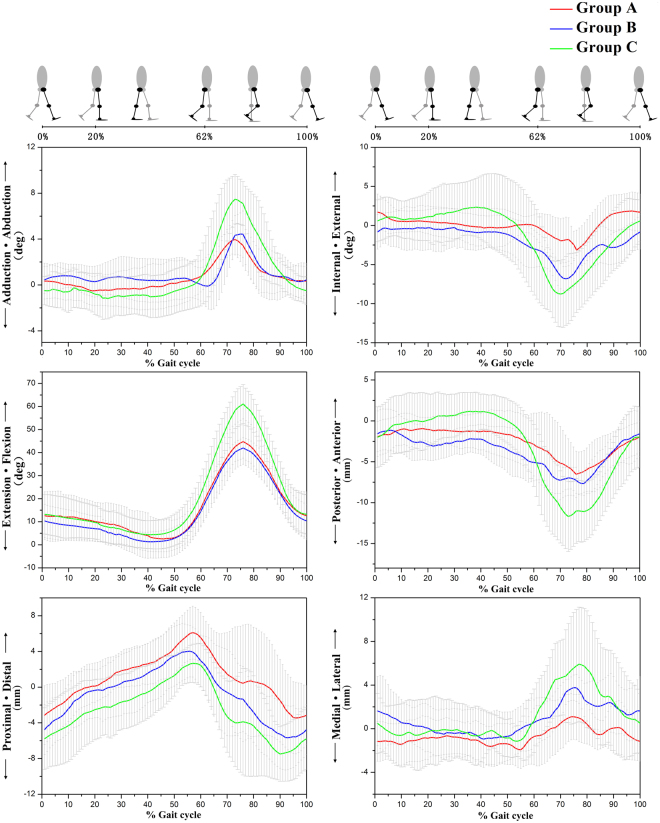
The three-dimensional knee joint rotation and translation of the subjects in different groups during treadmill gait. The thick solid lines represent the subjects’ average kinematics of the tibia relative to the femur, and the lines above and below the curves represent the standard deviation of the subjects. The red, blue, and green lines represent the average motion of the lateral discoid meniscus injury group, normal meniscus injury group, and healthy subject group, respectively.

Three-dimensional kinematic curves of knee joints are shown in Fig. 1, and five key motion parameters are listed in Table 2. During walking, knee joint movements were mainly exhibited in the sagittal plane. The lateral meniscus injury groups (Group A and Group B) showed decreases of approximately 14° to 16° in peak flexion angle compared to normal knee joints (Group C) (p < 0.05). The knees of patients in Group A and Group B had similar flexion-extension characteristics. The proximal-distal translation of the tibia in the swing phase of Group A was 2 mm smaller than that of Group B (p < 0.05) and 5 mm smaller than that of Group C (p < 0.05).

**Table 2:** Motion parameters in each group (mean ± SD).

	Group A	Group B	Group C
Maximum flexion angle in swing phase (deg.)	44.77 ± 9.87^*^	42.01 ± 4.44^#^	61.08 ± 8.44
Maximum internal angle in swing phase (deg.)	5.05 ± 1.38^*†^	7.07 ± 1.42^#^	10.78 ± 3.19
Maximum external angle in mid-stance phase (deg.)	1.18 ± 1.40	0.36 ± 2.44^#^	2.41 ± 3.11
Maximum distal translation in swing phase (mm)	4.08 ± 4.15^*^	6.50 ± 3.18	9.00 ± 3.68
Maximum lateral translation in swing phase (mm)	2.44 ± 1.90^*†^	4.80 ± 1.29^#^	7.22 ± 3.81

^*^P < 0.05 between Group A and Group C.

^#^P < 0.05 between Group B and Group C.

^†^P < 0.05 between Group A and Group B.

On the coronal plane, the peak tibia displacements in the swing phase of both injured groups were smaller than those of Group C (p < 0.05), and the maximum lateral tibial translation of Group A was 2 mm less than that in Group B (p < 0.05). In adduction-abduction rotation, a waveform-shaped curve denoted a similar pattern in the two groups of meniscus injury, and the peak abductions of the tibia in these two groups were significantly smaller than those in healthy knee joints (p < 0.05).

In terms of rotations on the transversal plane in the swing phase, the maximum internal rotation of the tibia of Group A was found to be decreased by 2° compared to that of Group B (p < 0.05), and 5° smaller than that of Group C (p < 0.05).

## Discussion

This study explores the kinematic characteristics of knees with lateral meniscus injury and lateral discoid meniscus injury, as well as those of healthy knees, using a portable optical tracking system. The results indicate that the two groups of patients with meniscus injury exhibited smaller ROMs than those with healthy knee joints. A few studies show that the symptoms of discoid meniscus injury are similar to those of normal meniscus injuries, such as pain, popping, and snapping. Chen *et al*.^20^ have reported the symptoms of discoid meniscus injury in both men and women. Popping and snapping were mainly found in males, and restricted ROM was mainly found in females. Restricted ROM was likely to be caused by the strain and pain of an injured meniscus. As a result, patients made adaptations to alleviate the discomfort caused by pain during walking.

In this study, knees with lateral discoid meniscus injury showed reduced internal rotation in the swing phase compared to healthy knees, which is consistent with the results of a study by Kengo *et al*.^21^. Previous studies indicate that healthy knees show external rotation in the terminal stance phase and internal rotation in the swing phase. Although the mechanism is not clearly understood, the authors speculate that these changes seem to be involved in the mild strains and movements of abnormal meniscus for self-adaptation. Compared to normal meniscus, the height and width of a discoid meniscus are increased. Meanwhile, the contact area between the meniscus and the cartilage is increased. The increased size of a meniscus led to the narrowing of the joint space. When a knee joint internally-externally rotates, non-physiological horizontal shear is formed. This abnormal shear limits the meniscal movement and initiates the destruction of the meniscus. However, restricted extension was not found in the terminal stance in knees with lateral discoid meniscus injury when compared to healthy knees, which contradicts Kengo’s findings^21^. This distinction was probably related to the varying walking conditions (over-ground walking in Kengo’s study versus treadmill walking in the current study). Besides, researchers have reported that subjects adopted a cautious gait on the treadmill compared to over-ground walking in response to the possible inherent balancing challenges imposed by treadmill walking^22^.

Based on the portable optical tracking system used in the current study, the kinematic differences between healthy knees, knees with lateral meniscus injury, and knees with lateral discoid meniscus injury were investigated. Based on the findings, a new method was proposed to assess *in vivo* dynamic function in knees with discoid meniscus injury. Traditional diagnosis mainly relies on physical and imaging examinations for the evaluation of knee status. The accuracy of physical examination ranges from 29% to 93%. It greatly relies on the experience and knowledge of clinicians^23^. In other words, in physical examinations, clinicians fail to quantitatively evaluate knee function. Through MRI scanning, which focuses on morphology, clinicians can obtain details about injured regions. Currently, MRI is the main diagnosis approach for discoid meniscus injury. However, functional movement cannot be evaluated using static medical imaging, such as MRI. The dynamic assessment of a knee joint will make a functional evaluation highly comprehensive, especially a postoperative evaluation following rehabilitation protocols. On the contrary, based on kinematics, changes in biomechanics will be found depending on different knee statuses. Using a 3D motion analysis system, Gao *et al*.^15^ measured the time-space parameters of the knee joints of 36 patients (twelve normal subjects, twelve patients with ACL injuries, and twelve patients with reconstructed ACLs) while they negotiated steps. The resulted showed that when the ACL was injured, the extension that occurred during step-climbing decreased while adduction and internal tibial rotation increased. Okazaki *et al*.^24^ assessed anterolateral rotatory stability in 36 patients before ACL reconstruction and 56 patients one year after ACL reconstruction via open magnetic resonance imaging (OMRI). The result showed that OMRI measures of the lateral tibial anterior of patients with ACL injury and ACL reconstructions were positively correlated to scores on pivot shift tests. In addition, OMRI can also be used to detect the contact area between the meniscus and the cartilage. The findings from the examinations listed above enable us to analyze kinematic and mechanical changes in knee joints during different functional movements. Kengo *et al*.^21^ used a ten-camera system based on 23 reflective markers placed on lower limbs and iliac crests to collect the 3D motion data about ten patients diagnosed with bilateral discoid lateral meniscus. This study simplified the structure of the traditional optical tracking equipment through the registration of bone marker points. In general, gait collection and analysis in laboratories requires too much labor and space and is inefficient. Instead, our portable motion capture system with two operators can be used to collect the kinematic information about a patient’s knee within five minutes, making it fast and convenient for clinicians to obtain quantitative information.

It should be noted there were some limitations in this study. First, the portable optical tracking system is sensitive to STA^25^. STA, which describes the relative motion between skin-mounted bone markers and the underlying bones, is mainly exhibited during the early-stance and mid-stance phases of a gait cycle. Second, only 42 subjects were recruited in this study. The sample size is not big enough to represent the general population and determine the kinematic characteristics of the knees of people in different populations. Third, the injury patterns of meniscal injury were not consistent in the different groups. Differing injury patterns may influence the kinematic characteristics of knees in different ways. Fourth, people’s movement patterns on treadmill may be different from those on the ground, despite the fact that some research reveals that only small differences in kinematic parameters can be found between treadmill and over-ground gait^26^. Fifth, we did not measure the contact pressure and area between the discoid meniscus and cartilage. These measurements require 2D-3D matching techniques or *in vitro* biomechanical studies for dynamic knee functional calculation. Sixth, the differences in the ROM of knee adduction/abduction and medial/lateral translation between groups were close to the measurement accuracy of optical tracking technique.

## Conclusion

This study reveals that maximum lateral tibial translation and maximum internal tibial rotation in knees with lateral discoid meniscus injury are significantly decreased compared to those with lateral meniscus injury. A new method was also proposed for the assessment of the kinematic characteristics of lateral discoid meniscus injury. It has potential for application in preoperative and postoperative evaluation.

## Methods

### Subjects Recruit

Fourteen patients diagnosed with unilateral lateral discoid meniscus injury (Group A), fourteen patients diagnosed with single-lateral meniscus injury (Group B), and fourteen healthy subjects (Group C) were recruited for this study. The healthy subjects had no history of knee pain, trauma, surgery, or obvious abnormal movement. All patients in Group A and Group B were diagnosed with Grade 3 injuries in their lateral meniscus via MRI (Magnetom Trio; Siemens Healthcare, Forchheim, Germany). The patients were confirmed to not have other injuries in their lower limbs via physical and imaging examination. This study was approved by the Ethical Committee of Guangzhou General Hospital of Guangzhou Military Command, and signed informed consent forms were obtained from all participants. It was conducted in accordance with the principles outlined in the Declaration of Helsinki. The subject demographics for each group (age, body mass index, and age) are provided in Table 3, and no statistically significant differences were found among groups.

**Table 3:** Subject demographics in each group (mean ± SD).

	Group A	Group B	Group C
Age (years)	26.1 ± 11.2	27.8 ± 10.7	27.0 ± 9.1
BMI (kg/m^2^)	19.9 ± 3.8	21.3 ± 1.6	21.4 ± 2.6
Sex (female/male)	9/5	8/6	6/8

### *In Vivo* Kinematic Assessment

**Figure 2:**
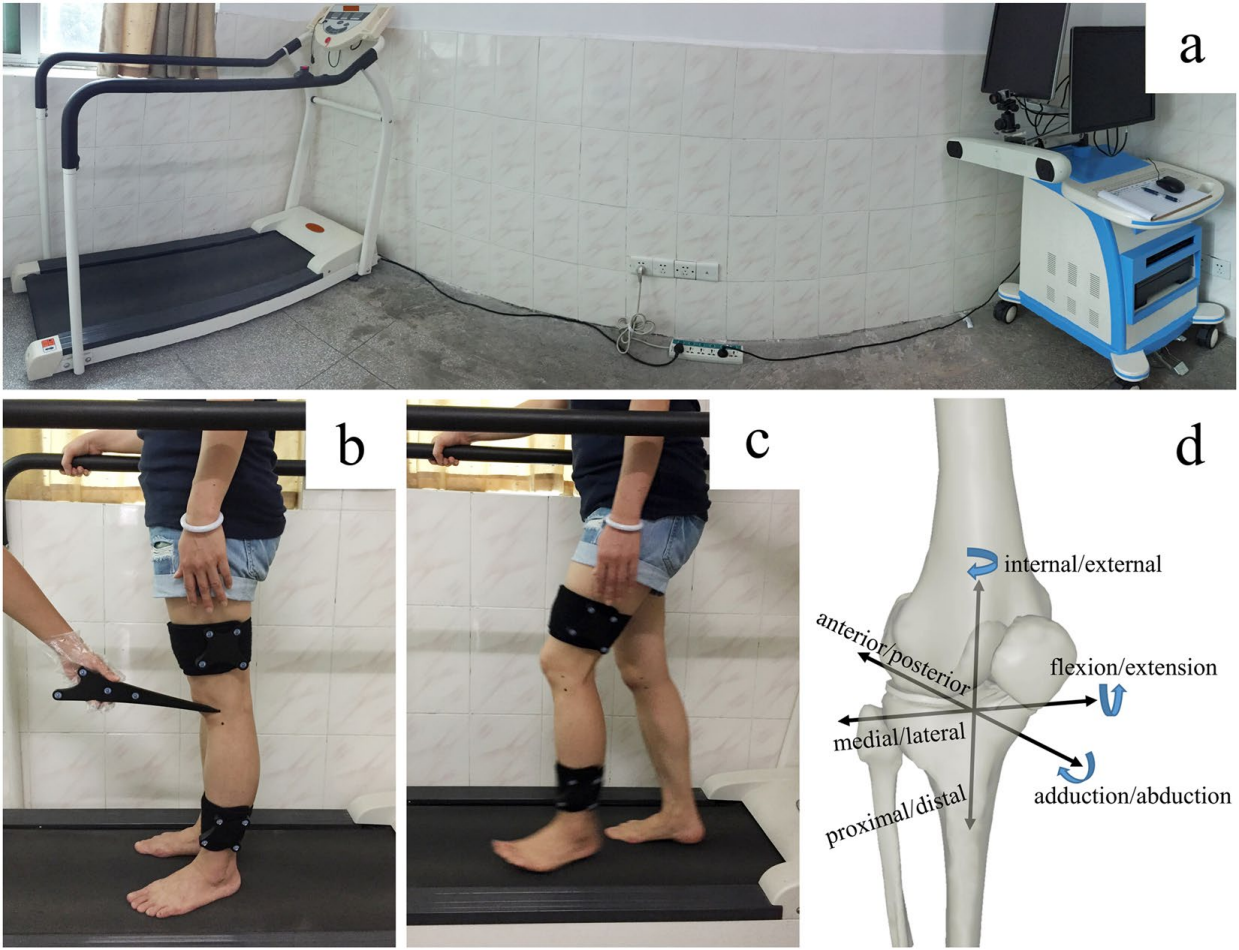
The portable optical tracking system and process of kinematics analysis. (**a**) The system has a working dimension of 1 m*2.5 m*1 m (length*width*height). (**b**) The identification of the femoral and tibial anatomic landmarks was performed using a handheld probe before the kinematic data was captured. (**c**) Kinematics data was collected while the subject was walking on a treadmill for 15 seconds. (**d**) Definition of local femoral and tibial coordinate systems.

A portable optical tracking system (Opti_Knee^®^, Shanghai Innomotion Company) was used to examine the kinematic characteristics of patients’ injured knees and healthy knees during walking (Fig. 2a). Firstly, spatial orientation was identified for bone landmarks with the assistance of handheld markers (Fig. 2b), including the greater trochanter of the femur, lateral and medial femoral condyle, lateral and medial tibial plateau, lateral fibular head, tibial tubercle, and medial and lateral malleolus. Two infrared inductors were fixed on the distal femurs and proximal tibias of respondents. Based on the bone landmarks in the system, three-dimensional coordinate systems of the femur and tibia were built. The rotation was defined as the tibia’s rotation along with the origin of the coordinate system in the femur. Similarly, displacement was defined as the tibia’s movement relative to the origin of the coordinate system in the femur.

The subjects walked on a treadmill for 2 minutes and chose comfortable speeds to ensure that their walking patterns were similar to when they walk on the ground (Fig. 2c). Kinematic data was collected in 15 seconds at a frequency of 60 frames per second. We performed a pilot study (see supplementary information) to validate the reliability and accuracy of the portable optical tracking system used in the current study. Each gait cycle was analyzed using a customized MATLAB procedure to normalize the gait cycle to 100 points. All 6 DOFs were analyzed, including adduction/abduction, internal/external, flexion/extension tibial rotation, anterior/posterior translation, proximal/distal translation, and medial/lateral tibial translation (Fig. 2d). Gait cycles were divided into stance phase and swing phase. The stance phase is defined as the time from heel strike to toe-off (0%–62% of the gait cycle), and the swing phase is defined as the time from toe-off until the next heel strike (63%–100% of the gait cycle). The ROM in 6 DOFs includes maximum distal translation in the swing phase, maximum lateral translation in the swing phase, maximum flexion angle in the swing phase, minimum external angle in the swing phase, maximum external angle in the mid-stance phase, and minimum distal translation in the mid-stance phase. After data collection, these parameters were calculated and compared among the three groups.

### Statistical Analysis

The kinematic characteristics of three groups were compared using one-way ANOVA for parametric variables, and the Mann–Whitney test was used for non-parametric variables. SPSS v13.0 (SPSS, Chicago, IL, USA) was used to execute the statistical analyses. The level of statistical significance was set at p < 0.05.

## Electronic supplementary material

Supplementary Information
