# Supplementary material for: Kinematic features in patients with lateral discoid meniscus injury during walking
Source: Sci Rep. 2018 Mar 22;8:5053. doi: 10.1038/s41598-018-22935-0 (PMC5864959; doi:10.1038/s41598-018-22935-0)
Supplement: Supplementary file 1 — Supplementary Information [file 41598_2018_22935_MOESM1_ESM.docx]

**Kinematic features in patients with** **lateral discoid meniscus injury during walking**

Zefeng Lin, Wenhan Huang, Limin Ma, Lingling Chen, Zhiqiang Huang, Xiaolong Zeng, Hong Xia, Yu Zhang

This supplementary information file contains some preliminary study results about the comparison between the motion analysis system and the biplanar fluoroscopy system in terms of validation.

**System Specification and Experiment Setup**

The Opti_Knee system is based on surgical navigation technology. Two high-speed inferred cameras were integrated into housing to fix the relative 3D space position of the cameras. The system captures the 3D position of points in 60 Hz, with a field of view of approximately 2 m x 2 m at a distance from 2 m to 3 m. The total space requirement of the system and testing area is about 10m^2^ Two marker sets are fixed to the thigh and shin, respectively. A digitizer calibrates specific patient bone landmark points: great trochanter, medial epicondyle, lateral epicondyle, medial tibia plateau, lateral tibia plateau, tibia tuberosity, fibula head, medial malleolus and lateral malleolus during an initial neutral standing position. The neutral standing position is also used as zero reference. Thirty subjects were recruited and required to walk on a treadmill and all gait cycles over 30 seconds and the position of marker sets was collected. The bony landmarks are calculated by the geometric relationship setup obtained from the initial positing. Six degree of freedom (6DOF) knee joint kinematics are calculated from the local coordinate systems of the femur and the tibia using the bony landmarks. The average, as well as standard deviation of knee kinematics from all gait cycles, is calculated through an automated program for a reliable analysis. The overall setup, training and experimental time is within 10 minutes.

**Validation of accuracy**

To validate the accuracy of the Opti_Knee system, a biplanar fluoroscopy system (Innomotion, Inc, Shanghai) was used as a gold standard for the calculation of 6DOF knee kinematics (Figure 1). The biplanar fluoroscopy system captured perspective bone images from two views and calculated joint kinematics through a semiautomatic 3D-2D matching process described in great details in the previous study [*Wang S et al. 2008 Spine, 33(11):E355-61*]. Without skin artifacts and directly assess bone movement, the biplanar fluoroscopy system is regarded as the most accurate noninvasive system with an accuracy of 0.3 mm and 0.6 degree. Flexion-extension cycles of the knee were recorded by both Opti_Knee and the biplanar fluoroscopy system. The measurement difference, the standard deviation of the difference between these two techniques were analyzed.


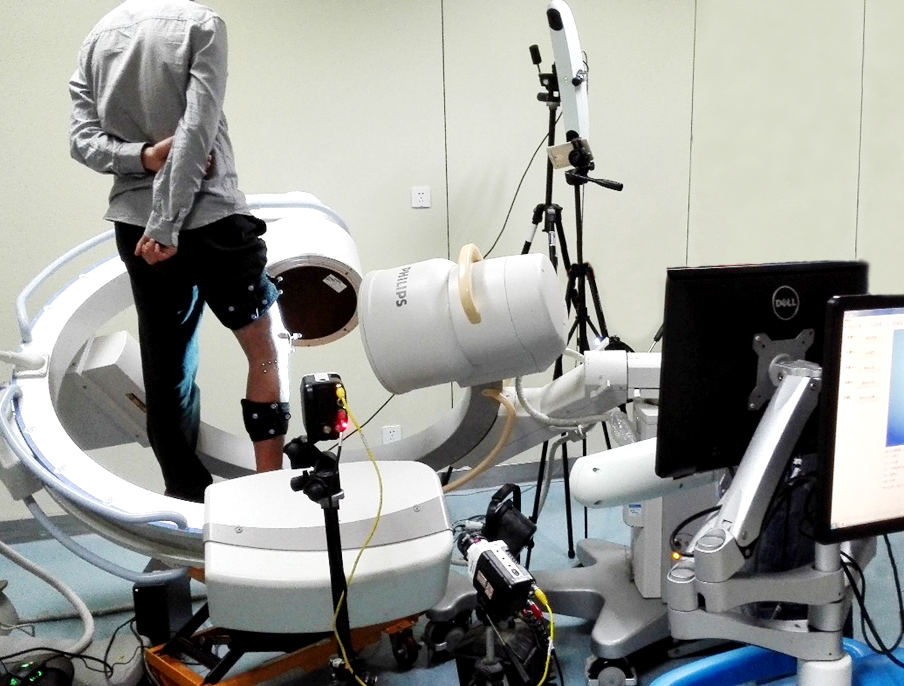


Figure 1: Flexion-extension of the knee captured by the biplanar fluoroscopy system

and the Opti_Knee system for validation.

**Validation of accuracy**

The absolute mean difference of rotational accuracy is 2.6 degree. The absolute mean difference of translation accuracy is 4.5 mm. (Table 1).

Table 1: Mean and standard deviation of rotational and translation accuracy during flexion. SD: standard deviation, AbAd: abduction/adduction, IE: internal/external, FE: flexion/extension, AP: anterior/posterior, DP: distal/proximal, ML: medial/lateral

|  |  | Mean(SD) |
| --- | --- | --- |
| Rotation (deg) | AbAd | -2.6(1.5) |
|  | IE | 0.5(4.0) |
|  | FE | -0.5(1.7) |
| Translation (mm) | AP | 3.5(2.0) |
|  | DP | 2.7(2.9) |
|  | ML | 4.5(2.3) |
